# Supplementary material for: Performance and feasibility of self-microsampling of capillary blood and saliva for serological testing of SARS-CoV-2
Source: PLoS One. 2025 Jul 11;20(7):e0327821. doi: 10.1371/journal.pone.0327821 (PMC12250565; doi:10.1371/journal.pone.0327821)
Supplement: S1 Appendix — (DOCX) [file pone.0327821.s001.docx]

# **Title:** **Performance and feasibility of self-microsampling of capillary blood and saliva for serological testing of SARS-CoV-2**

## **Authors**

Ivonne Morales^1,2*^, Josh Bueggeln^1^, Anna Denzler^3^, Vera Sonntag-Buck^4^, Kathleen Börner^4,5,6,7^, Petr Chlanda^6,8^, Lisa Koeppel^1^, Andreas Deckert^2^, Till Bärnighausen^2^, Michael Knop^3,9^, Claudia M. Denkinger^1,7^.

^1^ Department of Infectious Disease and Tropical Medicine, Heidelberg University Hospital, Heidelberg, Baden-Württemberg, Germany

^2^ Heidelberg Institute of Global Health, Heidelberg University Hospital, Heidelberg, Baden-Württemberg, Germany

^3^ Center for Molecular Biology of Heidelberg University (ZMBH), Heidelberg, Baden-Württemberg, Germany

^4^ Center for Infectious Diseases, Virology, Medical Faculty, Heidelberg University Hospital, Heidelberg, Baden-Württemberg, Germany

^5^ AskBio GmbH, Heidelberg, Baden-Württemberg, Germany.

^6^ BioQuant, Heidelberg University, Heidelberg, Baden-Württemberg, Germany.

^7^ German Center for Infection Research (DZIF), partner site Heidelberg, Heidelberg, Baden-Württemberg, Germany

^8^ Schaller Research Groups, Department of Infectious Diseases, Virology, Heidelberg University Hospital, Heidelberg, Baden-Württemberg, Germany

^9^ German Cancer Research Center (DKFZ), Heidelberg, Baden-Württemberg, Germany

***Corresponding author:**

E-mail: [ivonne.morales@uni-heidelberg.de](mailto:ivonne.morales@uni-heidelberg.de) (IM)

## **S1 Appendix.**

### **Study population**

We obtained a list of participants with evidence of a past SARS-CoV-2 infection and antibodies who consented to be informed about future studies. These participants were part of the BioBank Med V donor study, a study at Heidelberg University Hospital for the collection of human biomaterials and data for biomedical research. Participants had been recruited through a public advertisement in Heidelberg and the surrounding Rhein-Neckar district and were convalescent patients (at least three weeks post-symptom resolution). From this cohort, donors with a past SARS-CoV-2 infection and antibody evidence were recruited for our study. For the purposes of this study, a random number list was generated using the statistical program R to invite participants via phone or email. The first 50 participants from the random number list who expressed interest were invited to the study and consented at the clinic. Of the final 46 participants that provided a saliva sample, two were family members of BioBank Med V study participants who reported past SARS-CoV-2 infection and provided laboratory evidence of anti-SARS-CoV-2 antibodies.

Former participants of the seroprevalence study originated from a representative sample of the population. For this study, we obtained a list of seroprevalence study participants who had consented to receive information about future studies. These participants were contacted in the order in which we received their details and were invited to participate until we reached 102 participants. They were then invited to the clinic to provide consent. Out of the final 101 participants in the study, 99 had participated in the seroprevalence study and two were family members.

### **Challenges encountered during saliva collection**

Ten out of 46 participants reported facing challenges during saliva collection. Among these, not knowing how much saliva to provide/not knowing when the microsampler tips were full (40.0%) and not producing enough saliva (30.0%) were the most reported. Others favored a different saliva collection device having a Lolli-type format (20.0%) and one (10.0%) person was unaware he/she should abstain from eating/drinking prior to providing saliva. Improving the ability to recognize when the microsampler tips are full (n=2) and improving the design of the cartridge (n=1) were the suggestions received for improving the method.
