# Supplementary material for: Performance and feasibility of self-microsampling of capillary blood and saliva for serological testing of SARS-CoV-2
Source: PLoS One. 2025 Jul 11;20(7):e0327821. doi: 10.1371/journal.pone.0327821 (PMC12250565; doi:10.1371/journal.pone.0327821)
Supplement: S2 Appendix — (PDF) [file pone.0327821.s002.pdf]

Blood sample collection kit instructions*

Please read these instructions carefully before collecting your sample

Kit contents

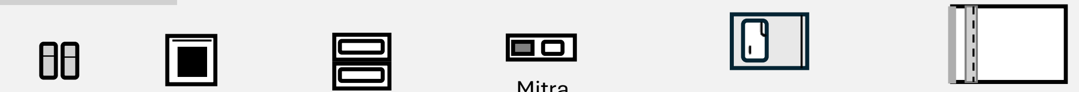

Lancet 2x | Gauze 1x | Bandage 2x | Mitra Cartridge 1x | Silver Specimen Bag 1x | Plastic Shipping Envelope 1x

First, unpack and prepare your kit contents

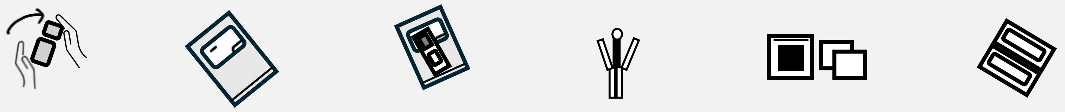

Twist off lancet caps | Tear open silver specimen bag | Remove cartridge | Open cartridge | Unwrap gauze | Unpeel back of bandages

step-by-step instructions

1. Wash your hands with warm, soapy water.

2. Warm your hands by rubbing them together.

3. Select the finger you will use to provide the blood sample. See illustration for recommended locations.

4. Prick your finger by laying hand on a hard surface. Position the lancet on the side of chosen fingertip. Apply firm pressure to end of lancet until a click is heard.

5. Wipe away first blood drop with the gauze. A blood drop may take up to 20 seconds to form. If needed, gently massage pricked finger upward until drop forms.

6. Collect sample by touching one of the two sampling tips to the blood drop. First, watch it turn fully red. Next, count 2 seconds. Last, slowly remove the tip from blood. Repeat this process with the second sampling tip.

make sure all sampler tips are filled correctly

Under-sampling occurs when: 1) The sampling tip is removed from the blood too soon. Touch tip to blood until no white remains. 2) Blood flow stops. If this happens, repeat steps 4-6 with same tip until it turns fully red.

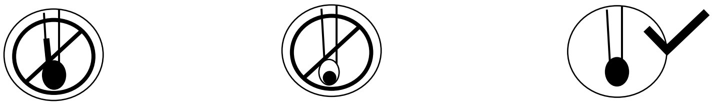

over-sampled | under-sampled | correctly sampled

Over-sampling occurs when blood is dripped onto the sampler tip from above. Always touch tip to blood drop as shown in step 6.

7. Set sampling device down on hard surface and apply bandage to your finger.

8. Close sampling device by lifting the sides to meet at the top. Press together until a click is heard.

9. Insert sampling device into the specimen bag and seal shut. Ensure the desiccant is still in the bag.

10. Insert specimen bag into plastic shipping envelope. Send to lab for processing as instructed.

*This instructional leaflet has been adapted from the original version (www.neoteryx.com) and is intended for illustrative purposes only.

Instructions for use*

Saliva collection

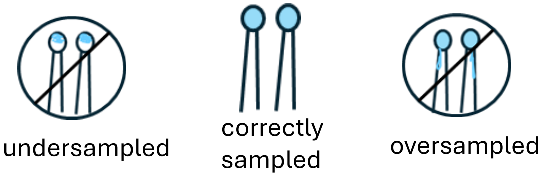

1. Prepare! See right-hand side.

2. Collect saliva in sputum cup.

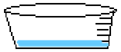

3. Replace lid on cup. Wash hands.

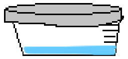

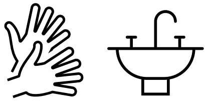

When sampling, be sure <u>NOT</u> to: fully submerge the sampling tips

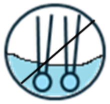

4. Touch the first sampling tip to the surface of the saliva. Watch it become fully saturated (~5 seconds). Then, count an additional 2 seconds and slowly remove it from the saliva. Repeat with other tip.

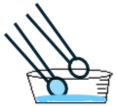

5. Close the cartridge.

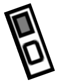

6. Insert cartridge into specimen bag, seal bag shut, and insert in shipping envelope.

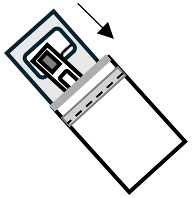

7. Discard saliva and cup safely.

Do this FIRST!

Remove cartridge from specimen bag.

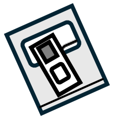

Do NOT remove desiccant.

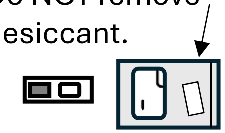

Open the Mitra cartridge by pulling apart the vented flaps.

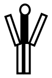

Do <u>NOT</u> remove sampling tips from cartridge.

Remove lid from provided saliva cup.

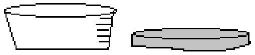

***This instructional leaflet has been adapted from the original version (www.neoteryx.com) and is intended for illustrative purposes only.**
