# Supplementary material for: Performance and feasibility of self-microsampling of capillary blood and saliva for serological testing of SARS-CoV-2
Source: PLoS One. 2025 Jul 11;20(7):e0327821. doi: 10.1371/journal.pone.0327821 (PMC12250565; doi:10.1371/journal.pone.0327821)
Supplement: S1 Table — (DOCX) [file pone.0327821.s003.docx]

**S1 Table. Participant demographic characteristics by antibody status.**

| Factors | Known antibody status, (n=48) | Unknown antibody status, (n=101) | Total (n=149) | *P* value |
| --- | --- | --- | --- | --- |
|  |  |  |  |  |
| Age, years |  |  |  | < .001^a^ |
| - Median (IQR) | 46.5 (20.5) | 58 (27) | 53 (25) |  |
| Sex, n (%) |  |  |  | .363^b^ |
| - Female | 19 (39.6%) | 48 (47.5%) | 67 (45.0%) |  |
| - Male | 29 (60.4%) | 53 (52.5%) | 82 (55.0%) |  |
| Highest general school qualification, n (%) |  |  |  | .434^b^ |
| - Upper secondary school | 31 (64.6%) | 67 (66.3%) | 98 (65.8%) |  |
| - Intermediate secondary school | 15 (31.2%) | 23 (22.8%) | 38 (25.5%) |  |
| - Lower secondary school | 2 (4.2%) | 10 (9.9%) | 12 (8.1%) |  |
| - Other | 0 (0.0%) | 1 (1.0%) | 1 (0.7%) |  |
| Vocational or technical training^c^, n (%) |  |  |  | NA |
| - Yes | 29 (100.0%) | 62 (100.0%) | 91 (100.0%) |  |
| - No | 0 (0.0%) | 0 (0.0%) | 0 (0.0%) |  |
| Higher education degree, n (%) |  |  |  | .222^b^ |
| - Yes | 27 (56.2%) | 46 (45.5%) | 73 (49.0%) |  |
| - No | 21 (43.8%) | 55 (54.5%) | 76 (51.0%) |  |
| Previous experience with a SARS-CoV-2 test based on a self-administered finger prick, n (%) |  |  |  | .392^b^ |
| - Yes | 2 (4.2%) | 8 (7.9%) | 10 (6.7%) |  |
| - No | 46 (95.8%) | 93 (92.1%) | 139 (93.3%) |  |
| Regular use of an at-home medical test requiring a self-administered finger prick, n (%) |  |  |  | .298^b^ |
| - Yes | 1 (2.1%) | 6 (5.9%) | 7 (4.7%) |  |
| - No | 47 (97.9%) | 95 (94.1%) | 142 (95.3%) |  |
| Lab working experience, n (%) |  |  |  | .002^b^ |
| - Yes | 12 (25.0%) | 7 (6.9%) | 19 (12.8%) |  |
| - No | 36 (75.0%) | 94 (93.1%) | 130 (87.2%) |  |
| Previous experience with a saliva based SARS-CoV-2 test^d^, n (%) |  |  |  | NA |
| - Yes | 9 (20.0%) | 0 | 9 (20.0%) |  |
| - No | 36 (80.0%) | 0 | 36 (80.0%) |  |
| Regular use of an at-home saliva collection device^e^, n (%) |  |  |  | NA |
| - Yes | 3 (6.7%) | 0 | 3 (6.7%) |  |
| - No | 42 (93.3%) | 0 | 42 (93.3%) |  |
| Vaccinated against SARS-CoV-2^f^, n (%) |  |  |  | .853^b^ |
| - Yes | 45 (95.7%) | 96 (95.0%) | 141 (95.3%) |  |
| Number of vaccine doses, n (%) |  |  |  | < .001^b^ |
| - 1 | 11 (24.4%) | 1 (1.0%) | 12 (8.5%) |  |
| - 2 | 23 (51.1%) | 67 (69.8%) | 90 (63.8%) |  |
| - 3 | 11 (24.4%) | 28 (29.2%) | 39 (27.7%) |  |
| - No | 2 (4.3%) | 5 (5.0%) | 7 (4.7%) |  |
| Past SARS-CoV-2 infection^g^, n (%) |  |  |  | < .001^b^ |
| - Yes | 48 (100.0%) | 5 (5.0%) | 53 (35.8%) |  |
| - No | 0 (0.0%) | 95 (95.0%) | 95 (64.2%) |  |
| Native language, n (%) |  |  |  | NA |
| - German | 44 (91.7%) | 95 (94.1%) | 139 (93.3%) |  |
| - English | 0 (0.0%) | 0 (0.0%) | 0 (0.0%) |  |
| - Other | 4 (8.3%) | 6 (5.9%) | 10 (6.7%) |  |

^a^ Linear Model ANOVA.

^b^ Pearson chi-square test.

^c^ Number of missing answers: known antibody status (n=19), unknown antibody status (n=39).

^d^ Out of 46 participants with known antibody status who provided saliva. Number of missing answers, n=1.

^e^ Out of 46 participants with known antibody status who provided saliva. Number of missing answers, n=1.

^f^ Number of missing answers, n=1 (known antibody status).

^g^ Number of missing answers, n=1 (unknown antibody status).

IQR= interquartile range.

NA= Not applicable.
