# Supplementary material for: Performance and feasibility of self-microsampling of capillary blood and saliva for serological testing of SARS-CoV-2
Source: PLoS One. 2025 Jul 11;20(7):e0327821. doi: 10.1371/journal.pone.0327821 (PMC12250565; doi:10.1371/journal.pone.0327821)
Supplement: S2 Table — (DOCX) [file pone.0327821.s004.docx]

**S2 Table. Univariate and multivariable linear regression analysis on log antibody titers measured in serum, a past SARS-CoV-2 infection, age, sex, and number of vaccine doses.**

|  |  | Serum antibody titers | | | | | |
| --- | --- | --- | --- | --- | --- | --- | --- |
|  |  | Univariate | | | Multivariable | | |
| Factors | **Total events, n=140 (%)** | **β^a^** | **95% CI^b^** | ***P* value** | **β^a^** | **95% CI^b^** | ***P* value** |
|  |  |  |  |  |  |  |  |
| *Age* | 140 (100) | 0.99 | (0.98-1.01) | .413 | 1.00 | (0.99-1.02) | .610 |
| *Sex* |  |  |  |  |  |  |  |
| Female (reference) | 65 (46.4) | 0 | - | - | 0 | - | - |
| Male | 75 (53.6) | 0.91 | (0.55-1.51) | .712 | 0.77 | (0.48-1.23) | .270 |
| *Past SARS-CoV-2 infection* |  |  |  |  |  |  |  |
| No (reference) | 92 (65.7) | 0 | - | - | 0 | - | - |
| Yes | 48 (34.3) | 3.55 | (2.19-5.78) | <.001 | 4.21 | (2.50-7.07) | <.001 |
| *Number of vaccine doses^c^* | 140 (100) | 1.10 | (0.70-1.71) | .677 | 1.45 | (0.96-2.21) | .079 |

^a^ exponentiated regression coefficient.

^b^ 95% confidence interval of the exponentiated regression coefficient.

^c^ The number of vaccine doses ranged between one and three.

S2 Table shows the respective coefficients and 95% CIs for the association between the log antibody titers in serum and having a past SARS-CoV-2 infection, age, sex, and the number of vaccine doses received. Results are shown for the univariate and multivariable models. Before adjustment, the results of the univariate regression analysis revealed that individuals with a past SARS-CoV-2 infection had serum titers 3.55 times higher (95% CI: 2.19-5.78) compared to those without an infection. This association was statistically significant (*P*<.001). After adjusting the model, the association between a past SARS-CoV-2 infection and antibody titers in serum increased in magnitude and remained statistically significant.
