# Supplementary material for: Performance and feasibility of self-microsampling of capillary blood and saliva for serological testing of SARS-CoV-2
Source: PLoS One. 2025 Jul 11;20(7):e0327821. doi: 10.1371/journal.pone.0327821 (PMC12250565; doi:10.1371/journal.pone.0327821)
Supplement: S3 Table — (DOCX) [file pone.0327821.s005.docx]

**S3 Table. Univariate and multivariable linear regression analysis on log antibody titers measured in capillary blood, a past SARS-CoV-2 infection, age, sex, and number of vaccine doses.**

|  |  | Capillary blood antibody titers—Health professional | | | | | |
| --- | --- | --- | --- | --- | --- | --- | --- |
|  |  | Univariate | | | Multivariable | | |
| Factors | **Total events, n=140 (%)** | **β^a^** | **95% CI^b^** | ***P* value** | **β^a^** | **95% CI^b^** | ***P* value** |
|  |  |  |  |  |  |  |  |
| *Age* | 140 (100) | 0.99 | (0.98-1.01) | .340 | 1.00 | (0.98-1.02) | .873 |
| *Sex* |  |  |  |  |  |  |  |
| Female | 65 (46.4) | 0 | - | - | 0 | - | - |
| Male | 75 (53.6) | 0.86 | (0.50-1.51) | .606 | 0.74 | (0.44-1.25) | .257 |
| *Past SARS-CoV-2 infection* |  |  |  |  |  |  |  |
| No (reference) | 92 (65.7) | 0 | - | - | 0 | - | - |
| Yes | 48 (34.3) | 3.33 | (1.93-5.76) | <.001 | 3.92 | (2.18-7.04) | <.001 |
| *Number of vaccine doses^c^* | 140 (100) | 1.15 | (0.71-1.88) | .562 | 1.52 | (0.95-2.44) | .079 |
|  |  | **Capillary blood antibody titers—Participants** | | | | | |
|  |  | Univariate | | | Multivariable | | |
| Factors | **Total events, n=140 (%)** | **β^a^** | **95% CI^b^** | ***P* value** | **β^a^** | **95% CI^b^** | ***P* value** |
|  |  |  |  |  |  |  |  |
| *Age* | 140 (100) | 0.99 | (0.97-1.00) | .191 | 1.00 | (0.98-1.02) | .878 |
| *Sex* |  |  |  |  |  |  |  |
| Female (reference) | 65 (46.4) | 0 | - | - | 0 | - | - |
| Male | 75 (53.6) | 0.82 | (0.47-1.44) | .492 | 0.70 | (0.42-1.18) | .181 |
| *Past SARS-CoV-2 infection* |  |  |  |  |  |  |  |
| No (reference) | 92 (65.7) | 0 | - | - | 0 | - | - |
| Yes | 48 (34.3) | 3.58 | (2.08-6.17) | <.001 | 4.19 | (2.35-7.48) | <.001 |
| *Number of vaccine doses^c^* | 140 (100) | 1.18 | (0.72-1.92) | .502 | 1.60 | (1.00-2.55) | .048 |

^a^ exponentiated regression coefficient.

^b^ 95% confidence interval of the exponentiated regression coefficient.

^c^ The number of vaccine doses ranged between one and three.

S3 Table shows the respective coefficients and 95% CIs for the association between the log antibody titers in capillary blood and having a past SARS-CoV-2 infection, age, sex, and the number of vaccine doses received. Results are shown for the univariate and multivariable models. Statistically significant associations were observed between the antibody titers in capillary blood and having a past SARS-CoV-2 infection in the unadjusted model. On average, the antibody titers in capillary blood collected by health professionals were 3.33 times higher (95% CI: 1.93-5.76, *P*<.001), while those collected by study participants were 3.58 times higher (95% CI: 2.08-6.17, *P*<.001) in individuals with a past SARS-CoV-2 infection. After adjustment, the association between a past SARS-CoV-2 infection and antibody titers increased in magnitude and remained statistically significant. Furthermore, a higher number of vaccine doses was found to be positively associated with antibody titers in capillary blood collected by the study participants in the adjusted model.
