# Supplementary material for: Performance and feasibility of self-microsampling of capillary blood and saliva for serological testing of SARS-CoV-2
Source: PLoS One. 2025 Jul 11;20(7):e0327821. doi: 10.1371/journal.pone.0327821 (PMC12250565; doi:10.1371/journal.pone.0327821)
Supplement: S1 Fig — (DOCX) [file pone.0327821.s006.docx]

### Participant confidence during the self-sampling procedure

###
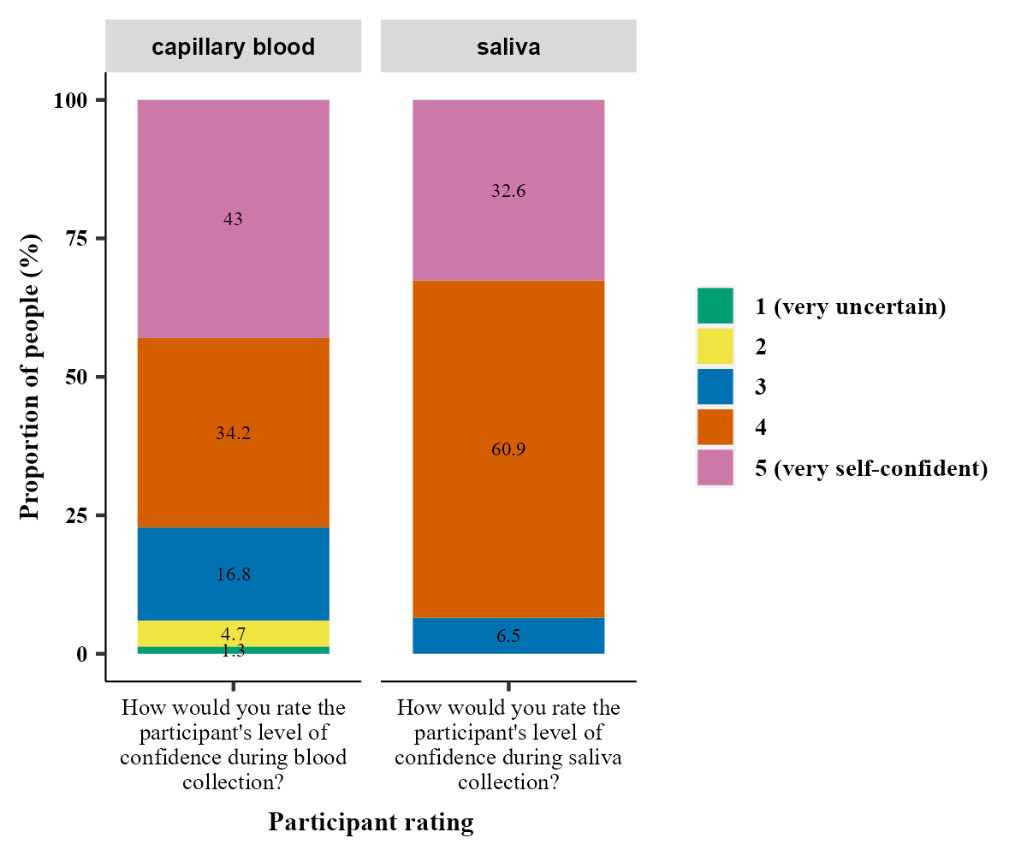


**S1 Fig. Observer ratings of participant confidence during capillary blood or saliva collection.** After observing the complete self-sampling procedure, the observer assessed the participant’s level of confidence during self-sampling for capillary blood or saliva using a Likert-type scale ranging from 1 ("very uncertain") to 5 ("very self-confident").
