# Supplementary material for: Performance and feasibility of self-microsampling of capillary blood and saliva for serological testing of SARS-CoV-2
Source: PLoS One. 2025 Jul 11;20(7):e0327821. doi: 10.1371/journal.pone.0327821 (PMC12250565; doi:10.1371/journal.pone.0327821)
Supplement: S2 Fig — (DOCX) [file pone.0327821.s007.docx]

### User experience

On a scale from 1 (easy) to 5 (difficult), participants rated the ease of collecting capillary blood (n=149) and/or saliva (46/149), including the use of the microsampler, the clarity of the instructions provided (written, video, or both) as well as the ease of self-sampling by the layperson (S2-S3 Figs). Regarding capillary blood collection, self-administering a finger prick or the use of the microsampler was easy for 81.8% (121/148) and 53.4% (78/146) of participants, respectively. Online video instructions were easier (87.8%, 122/139) to understand compared to the written instructions provided by the manufacturer (57.6%, 80/139) (S2 Fig). Overall, the self-finger prick was considered easy (score=1, 37.0%) or relatively easy (score=2, 45.2%) for the layperson. The use of the microsampler by the layperson received similar scores (score=1, 36.1% and score=2, 45.8%) (S2 Fig).


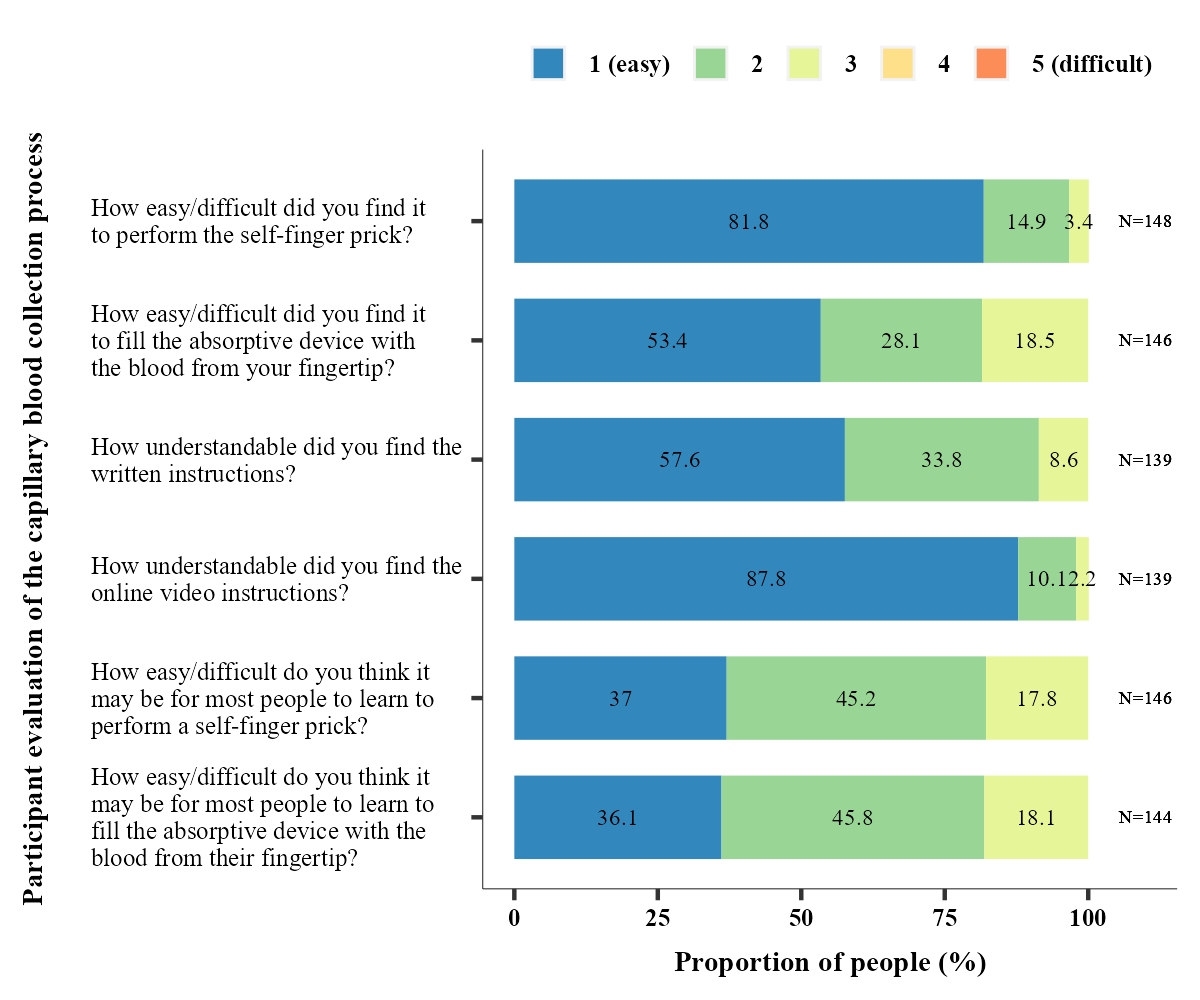


**S2 Fig. Participant rating of the capillary blood self-sampling procedure.** Participants used a Likert-type scale ranging from 1 ("easy") to 5 ("difficult") to rate the ease of different steps in the self-sampling procedure for capillary blood. N indicates the total number of respondents.
