# Supplementary material for: Performance and feasibility of self-microsampling of capillary blood and saliva for serological testing of SARS-CoV-2
Source: PLoS One. 2025 Jul 11;20(7):e0327821. doi: 10.1371/journal.pone.0327821 (PMC12250565; doi:10.1371/journal.pone.0327821)
Supplement: S4 Table — (DOCX) [file pone.0327821.s008.docx]

The self-reported mean time to complete the entire capillary blood collection procedure was 6.6 min (SD, ±3.6 min). When examining the influence of various demographic factors (e.g., age, sex, education, prior experience with self-testing) on the time required for sample collection, it was observed that individuals who rated the ease of the microsampler use for laypersons as ‘relatively easy’ or ‘not easy, not difficult’ required on average 1.37 and 2.54 extra minutes, respectively, for sample collection compared to those who rated the procedure as easy. These associations were statistically significant (*P*=.040 and *P*=.003, respectively) (S4 Table).

**S4 Table.** **Demographic factors associated with time to collection of a capillary blood sample.**

| Factor |  | Number of events (n=137) | Mean time (SD) | Coefficient (95% CI, P value) (univariable) |
| --- | --- | --- | --- | --- |
|  |  |  |  |  |
| **Age, years** |  | 137 (100.0) | 6.4 (3.6) | 0.02 (-0.02 to 0.05, *P*=.383) |
| **Sex** | Female | 60 (43.8) | 6.8 (4.0) | - |
|  | Male | 77 (56.2) | 6.1 (3.2) | -0.64 (-1.86 to 0.58, *P* =.300) |
| **Education^a^** |  | 137 (100.0) | 6.4 (3.6) | 0.31 (-0.60 to 1.23, *P*=.498) |
| **Any prior experience with self-testing or work at a laboratory** | No | 108 (78.8) | 6.6 (3.5) | - |
|  | Yes | 29 (21.2) | 5.8 (3.9) | -0.80 (-2.28 to 0.68, *P* =.287) |
| **Ease self-administering a finger prick** | Easy | 115 (83.9) | 6.3 (3.5) | - |
|  | Relatively easy | 18 (13.1) | 6.4 (4.0) | 0.04 (-1.75 to 1.84, *P* =.964) |
|  | Not easy, not difficult | 4 (2.9) | 8.8 (4.8) | 2.40 (-1.20 to 6.01, *P* =.190) |
| **Ease of microsampler use** | Easy | 74 (54.0) | 6.3 (3.6) | - |
|  | Relatively easy | 39 (28.5) | 6.5 (3.5) | 0.16 (-1.25 to 1.57, *P* =.818) |
|  | Not easy, not difficult | 24 (17.5) | 6.8 (3.7) | 0.45 (-1.22 to 2.13, *P* =.593) |
| **Ease of the finger prick for laypersons** | Easy | 53 (38.7) | 6.0 (3.5) | - |
|  | Relatively easy | 60 (43.8) | 6.5 (3.3) | 0.45 (-0.89 to 1.78, *P* =.508) |
|  | Not easy, not difficult | 24 (17.5) | 7.2 (4.4) | 1.19 (-0.55 to 2.93, *P* =.179) |
| **Ease of the microsampler for laypersons** | Easy | 50 (36.5) | 5.3 (3.1) | - |
|  | Relatively easy | 62 (45.3) | 6.7 (3.4) | 1.37 (0.06 to 2.68, *P* =.040) |
|  | Not easy, not difficult | 25 (18.2) | 7.9 (4.4) | 2.54 (0.85 to 4.23, *P* =.003) |

^a^ Includes one individual who selected 'other' as their education level and mentioned having a vocational degree. This person was categorized under lower secondary school, assuming that they would have needed at least a lower secondary degree to pursue a vocational qualification. CI: Confidence interval. SD: Standard deviation.
