# Supplementary material for: Performance and feasibility of self-microsampling of capillary blood and saliva for serological testing of SARS-CoV-2
Source: PLoS One. 2025 Jul 11;20(7):e0327821. doi: 10.1371/journal.pone.0327821 (PMC12250565; doi:10.1371/journal.pone.0327821)
Supplement: S5 Table — (DOCX) [file pone.0327821.s009.docx]

Out of the 149 participants that self-sampled for capillary blood, 25 noted the following limitations that could have hindered their performance: nervousness (28.0%), not having enough blood at the site of the finger prick (20.0%), insecurity (12.0%), visual impairment (8.0%), and problems with trembling/arthritis/cognition (16.0%) were the most frequently reported among them (S5 Table).

**S5 Table. Difficulties encountered during capillary blood collection, or which could make collection difficult for others.**

| Reason | Number (%) |
| --- | --- |
|  |  |
| Nervousness/anxiety | 7 (28%) |
| Not enough blood produced after the finger prick/having cold fingers | 5 (20%) |
| Insecurity | 3 (12%) |
| Trembling/Arthritis/cognitive problems | 4 (16%) |
| Visual impairment | 2 (8%) |
| Trouble opening the packaging material | 1 (4%) |
| Underestimating the video instructions/the video went too fast/prefers written instructions in general | 3 (12%) |
| Total | 25 (100%) |
