# Supplementary material for: Performance and feasibility of self-microsampling of capillary blood and saliva for serological testing of SARS-CoV-2
Source: PLoS One. 2025 Jul 11;20(7):e0327821. doi: 10.1371/journal.pone.0327821 (PMC12250565; doi:10.1371/journal.pone.0327821)
Supplement: S6 Table — (DOCX) [file pone.0327821.s010.docx]

Suggestions (feedback from n=41 participants) for improving self-collection included simplifying the manufacturer-provided written instructions (24.4%), a better description of the kit contents (14.6%), simplifying the opening of the cartridge and specimen bags (12.2%), clearer instructions for filling the microsampler tips (9.8%), and easier handling of the microsampler tips (7.3%) (S6 Table).

**S6 Table. Participants’ feedback on capillary blood collection.**

| Comment | Number (%) |
| --- | --- |
|  |  |
| Simplify the manufacturer’s instruction leaflet | 10 (24.4%) |
| Improve the description of the kit contents | 6 (14.6%) |
| Simplify the opening of the cartridge and packaging material | 5 (12.2%) |
| Clearer instructions for the use of the microsampler are needed. | 4 (9.8%) |
| The design of the microsampler tips could be improved (i.e., the tips are too close to each other which makes handling challenging; the tips could be packaged individually) | 3 (7.3%) |
| Provide recommendations to improve the blood flow | 2 (4.9%) |
| Sanitation concerns (i.e., the kit/packaging materials generate too much waste; advice that an absorbent material may be required in case there is too much blood) | 2 (4.9%) |
| Slow down the video | 2 (4.9%) |
| Other (i.e., could consider adding a QR code to the video on the written instructions leaflet, the procedure should be analogous to testing for your own blood sugar levels, etc.) | 5 (12.2%) |
| Ambiguous comment | 2 (4.9%) |
| Total | 41 (100%) |
