# Supplementary material for: Performance and feasibility of self-microsampling of capillary blood and saliva for serological testing of SARS-CoV-2
Source: PLoS One. 2025 Jul 11;20(7):e0327821. doi: 10.1371/journal.pone.0327821 (PMC12250565; doi:10.1371/journal.pone.0327821)
Supplement: S3 Fig — (DOCX) [file pone.0327821.s011.docx]

Among participants who also provided a saliva sample (46/149), 52.2% found saliva collection easy (score=1), while a higher proportion (84.8%) found the self-finger prick to be easy (S3 Fig). A similar trend was observed with the use of the microsampler, with 37.0% finding saliva collection with the microsampler easy compared to 52.2% for capillary blood collection. In contrast, the written instructions for saliva collection were easier to understand than those for taking capillary blood (70.0% and 64.3%, respectively). Additionally, self-sampling for saliva was faster than self-sampling for capillary blood (mean: 4.5 min, SD, ±2.4 min) and it was considered easier to learn by laypersons than the self-finger prick (51.1% and 24.4%, respectively) (S3 Fig).

**
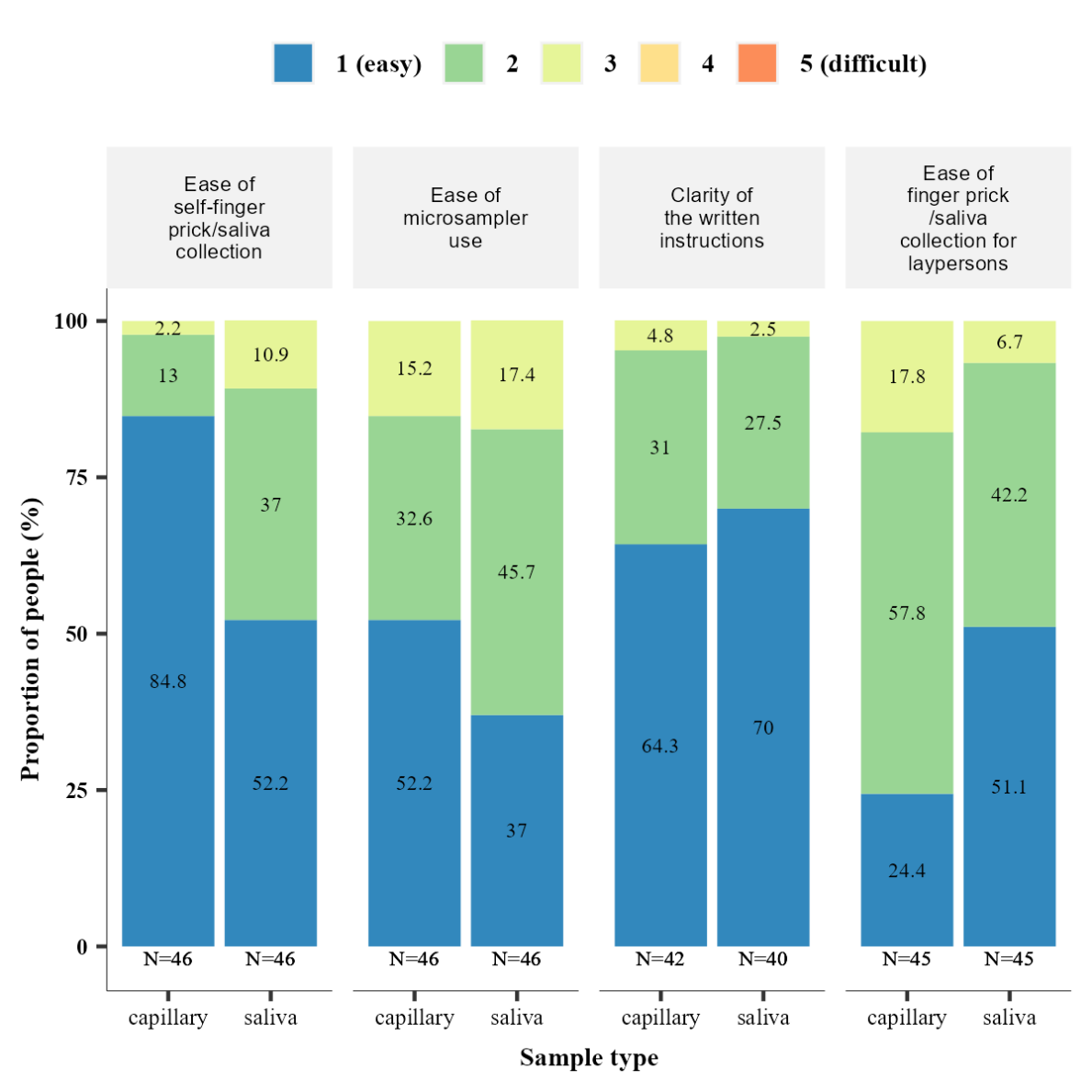
**

**S3 Fig.** **Participant rating of the saliva self-sampling procedure.** Participants used a Likert-type scale ranging from 1 ("easy") to 5 ("difficult") to rate the ease of different steps in the self-sampling procedure for saliva. The results presented are compared with those from participants who, in addition to providing a saliva sample, also self-collected capillary blood. N indicates the total number of respondents.
