# Supplementary material for: Performance and feasibility of self-microsampling of capillary blood and saliva for serological testing of SARS-CoV-2
Source: PLoS One. 2025 Jul 11;20(7):e0327821. doi: 10.1371/journal.pone.0327821 (PMC12250565; doi:10.1371/journal.pone.0327821)
Supplement: S7 Table — (DOCX) [file pone.0327821.s012.docx]

The estimated mean difference in time required for saliva collection for individuals who rated the self-collection procedure as ‘not easy, not difficult’ for laypersons was 3.38 min (*P*=.020) (S7 Table).

**S7 Table. Demographic factors associated with time to collection of a saliva sample.**

| Factor |  | Number of events (n=44) | Mean time (SD) | Coefficient (95% CI, P value) (univariable) |
| --- | --- | --- | --- | --- |
|  |  |  |  |  |
| **Age, years** |  | 44 (100.0) | 4.5 (2.4) | 0.03 (-0.03 to 0.09, *P*=.278) |
| **Sex** | Female | 17 (38.6) | 4.8 (2.5) | - |
|  | Male | 27 (61.4) | 4.3 (2.3) | -0.43 (-1.92 to 1.06, *P* =.562) |
| **Education** |  | 44 (100.0) | 4.5 (2.4) | -0.75 (-1.99 to 0.49, *P* =.229) |
| **Any prior experience with self-testing involving saliva collection** | No | 34 (77.3) | 4.5 (2.6) | - |
|  | Yes | 10 (22.7) | 4.4 (1.6) | -0.13 (-1.87 to 1.61, *P* =.881) |
| **Ease of saliva collection** | Easy | 24 (54.5) | 4.2 (2.5) | - |
|  | Relatively easy | 15 (34.1) | 4.9 (2.2) | 0.68 (-0.91 to 2.28, *P* =.393) |
|  | Not easy, not difficult | 5 (11.4) | 4.4 (2.6) | 0.15 (-2.24 to 2.54, *P* =.900) |
| **Ease of microsampler use** | Easy | 16 (36.4) | 3.9 (1.9) | - |
|  | Relatively easy | 20 (45.5) | 5.0 (2.5) | 1.11 (-0.49 to 2.71, *P* =.168) |
|  | Not easy, not difficult | 8 (18.2) | 4.2 (3.0) | 0.31 (-1.76 to 2.38, *P* =.762) |
| **Ease of saliva collection procedure for laypersons** | Easy | 22 (50.0) | 4.0 (2.1) | - |
|  | Relatively easy | 19 (43.2) | 4.7 (2.4) | 0.73 (-0.70 to 2.16, *P* =.309) |
|  | Not easy, not difficult | 3 (6.8) | 7.3 (2.5) | 3.38 (0.57 to 6.19, *P* =.020) |

CI: Confidence interval. SD: Standard deviation.
