# Supplementary material for: Performance and feasibility of self-microsampling of capillary blood and saliva for serological testing of SARS-CoV-2
Source: PLoS One. 2025 Jul 11;20(7):e0327821. doi: 10.1371/journal.pone.0327821 (PMC12250565; doi:10.1371/journal.pone.0327821)
Supplement: Attachment — Submitted filename: PONE-D-24-58079-reviewer view-gtb.docx [file pone.0327821.s014.docx]

I read the manuscript with great interest. The study focuses on the choice of sample and sampling methods, which are very critical in clinical laboratory diagnosis, in particular during a pandemic, quality laboratory diagnosis, and easy-to-collect samples are essential parts of pandemic containment. Thus, as part of preparedness for the coming pandemic, the study is important, despite they have employed expensive technology that will make it difficult for resource-limited settings to benefit from the technology.

1. The authors used high-throughput electrochemiluminescence technology, a very expensive one, for the antibody analysis. How is this feasible for scalability?
2. How could the authors talk about the cost-effectiveness of their method without comparing the prices of different versions of sample collection methods?
3. When it comes to result interpretation, my major concern comes from the study included small study participants, and they have a mixed population with natural infection and vaccination, which the authors also acknowledged. In addition to what they did, I expect the authors to disaggregate the study participants into three those with natural infection with no vaccination, those Vaccinated but not infected, and both infected and vaccinated, and re-do the analysis and see the test agreements between the groups.
