# Supplementary material for: Performance and feasibility of self-microsampling of capillary blood and saliva for serological testing of SARS-CoV-2
Source: PLoS One. 2025 Jul 11;20(7):e0327821. doi: 10.1371/journal.pone.0327821 (PMC12250565; doi:10.1371/journal.pone.0327821)
Supplement: Attachment — Submitted filename: Response_to_Reviewers.docx [file pone.0327821.s016.docx]

Reviewers' comments:

Reviewer's Responses to Questions

**Comments to the Author**

1. Is the manuscript technically sound, and do the data support the conclusions?

The manuscript must describe a technically sound piece of scientific research with data that supports the conclusions. Experiments must have been conducted rigorously, with appropriate controls, replication, and sample sizes. The conclusions must be drawn appropriately based on the data presented.

Reviewer #1: Yes

Reviewer #2: Yes

Reviewer #3: Yes

2. Has the statistical analysis been performed appropriately and rigorously?

Reviewer #1: Yes

Reviewer #2: Yes

Reviewer #3: Yes

3. Have the authors made all data underlying the findings in their manuscript fully available?

The [PLOS Data policy](http://track.editorialmanager.com/CL0/http:%2F%2Fwww.plosone.org%2Fstatic%2Fpolicies.action%23sharing/1/010f0194669246e4-de2630f1-3362-4fbe-90e7-77ed05e9716b-000000/RlzrrPTPL92MXU1aVsaaKjcQGvL0sptxsEd3KEMvxtI=193) requires authors to make all data underlying the findings described in their manuscript fully available without restriction, with rare exception (please refer to the Data Availability Statement in the manuscript PDF file). The data should be provided as part of the manuscript or its supporting information, or deposited to a public repository. For example, in addition to summary statistics, the data points behind means, medians and variance measures should be available. If there are restrictions on publicly sharing data—e.g. participant privacy or use of data from a third party—those must be specified.

Reviewer #1: No

Reviewer #2: Yes

Reviewer #3: Yes

4. Is the manuscript presented in an intelligible fashion and written in standard English?

PLOS ONE does not copyedit accepted manuscripts, so the language in submitted articles must be clear, correct, and unambiguous. Any typographical or grammatical errors should be corrected at revision, so please note any specific errors here.

Reviewer #1: Yes

Reviewer #2: Yes

Reviewer #3: Yes

**5. Review Comments to the Author**

Please use the space provided to explain your answers to the questions above. You may also include additional comments for the author, including concerns about dual publication, research ethics, or publication ethics. (Please upload your review as an attachment if it exceeds 20,000 characters)

**Reviewer #1:** Evaluation of the Manuscript "Sensitive and Feasible SARS-CoV-2 Antibody Detection from Blood and Saliva Self-Microsamples" Submitted to PLOS ONE for Publication Consideration

Volumetric absorptive microsampling (VAMS) has emerged as a valuable tool for serosurveillance and serodiagnosis during the COVID-19 pandemic. VAMS allows for remote, at-home sample collection, enabling large-scale serosurveillance studies that revealed higher SARS-CoV-2 seroprevalence than initially expected. This technique offers several advantages, including ease of use, minimal invasiveness, room temperature storage, and fixed volume absorption, which improves accuracy and reduces hematocrit effects.

Given the abundance of evidence demonstrating the use of VAMS in serosurveillance studies, including SARS-CoV-2, this study provides limited new information to the literature. Additionally, the sample size is too small, and most study participants are educated, as noted by the authors, which compromises the feasibility evaluation. Despite its limitations, this study may add useful information on the use of professional videos for sample collection using VAMS. Below are my major comments for the authors to consider to enhance the importance of their study.

Major Comments:

1. Title Revision: The title should reflect what the authors have done and found. I suggest modifying it to: Utility and Feasibility of Self-Sampling of Capillary Blood Using a Mitra Volumetric Absorptive Microsampling (VAMS) Device for Serological Testing of SARS-CoV-2.

We thank the reviewer for this helpful suggestion and agree that the title merits a revision to reflect a major aspect of this study. Since *clinical utility* has a specific meaning within the diagnostic value cycle—referring to a diagnostic test’s usefulness in improving patient outcomes, informing clinical decision-making, and optimizing healthcare resources—we prefer to use the term *performance* instead of *utility*.

The manuscript title has been changed to ‘Performance and feasibility of self-microsampling of capillary blood and saliva for serological testing of SARS-CoV-2.’

2. Objective Clarification (Lines 64-66): The objective should be modified to accurately reflect the study's aim. The sensitivity pertains to the assay, which in this case is the electrochemiluminescence-based Roche Elecsys anti-SARS-CoV-2 immunoassay. If the authors used VAMS for saliva collection, the objective statement and title should reflect this. The discussion should also support this with relevant results, as limited data exist on the use of VAMS for saliva collection, transportation, and storage in serosurveillance studies.
We thank the reviewer for this comment. We have included ‘saliva’ in the revised study title. Lines 77-79 include saliva as part of the objectives of the study: “In this study, we aimed to evaluate the performance and feasibility of the Mitra VAMS device for self-collection of finger prick blood and saliva to measure anti-SARS-CoV-2 antibodies using a high-throughput electrochemiluminescence-based SARS-CoV-2 spike (S) antibody assay.”

Indeed, limited data exist regarding the use of VAMS with saliva as a sample. We have now extended our discussion on this matter to highlight that valid antibody results were also obtained in saliva samples collected with the microsampler device. We also note that using the same type of device for both sample types (saliva and capillary blood) offers convenience as it can streamline sample processing. Moreover, we add evidence indicating that while antibody stability at various storage conditions and temperatures has been shown for influenza antibodies in VAMS-collected capillary blood, there is evidence of the feasibility of VAMS with oral fluids for the detection of stimulant drugs even under varying temperature, humidity, and light conditions. Finally, we extend this discussion with additional suggestions on how to improve antibody detection from saliva. This has been revised in the manuscript discussion (lines: 310-312; 315-320; 331-332; 335-339; 343-352).

3. Terminology Adjustment (Line 198): Instead of "Sensitivity of capillary, saliva, and serum samples," it should be "Agreement between saliva or capillary blood collected using the VAMS device," to align with the clarification above.

We thank the reviewer for the valuable comment and have adjusted the section title to ‘Agreement between paired serum and capillary blood or saliva samples.’ The term sensitivity has also been adjusted to ‘agreement’ or ‘positive percent agreement’ throughout the manuscript text and it is shown in a revised version of manuscript Table 2.

Table 2. Positive percent agreement between paired serum and capillary blood, or saliva samples from individuals with known antibody status.

| **Roche Elecsys anti-SARS-CoV-2 S RBD** | | | | | |
| --- | --- | --- | --- | --- | --- |
|  | **Serum (n=48)** | | | |  |
| Known antibody status |  | **Positive** | **Negative** | **Total** | **Positive percent (%) agreement** |
|  |  |  |  |  |  |
| **Health professional**  **capillary blood (n=48)^a^** | **Positive** | 48 | 0 | 48 | 100 (92.6-100) |
|  | **Negative** | 0 | 0 | 0 |  |
|  | **Total** | 48 | 0 | 48 |  |
|  |  | | | |  |
| **Participants**  **capillary blood (n=48)^b^** | **Positive** | 48 | 0 | 48 | 100 (92.6-100) |
|  | **Negative** | 0 | 0 | 0 |  |
|  | **Total** | 48 | 0 | 48 |  |
|  |  |  |  |  |  |
| **Participants**  **saliva (n=46)^c^** | **Positive** | 44 | 0 | 44 | 95.7 (85.2-99.5) |
|  | **Negative** | 2 | 0 | 2 |  |
|  | **Total** | 46 | 0 | 46 |  |

^a^ Samples collected by a health professional.

^b^ Samples self-collected by participants.

^c^ Saliva specimens were obtained from 46 of the 48 participants with known antibody status. The respective, 46 paired serum samples were used to measure positive percent agreement.

To help distinguish this agreement from the use of Cohen’s kappa, we have further distinguished between intra- and inter-rater agreement and this distinction has also been specified in the methods section.

In addition, since sensitivity/specificity are not assessed in this study, the following sentence was removed from the discussion ‘Pre-pandemic or negative patient samples were unavailable for this study; therefore, specificity could not be assessed.’
4. Comparison Enhancement: The study would have more impact if it compared different VAMS devices or compared VAMS-based capillary blood findings with conventional dried blood spots (DBS).

We thank the reviewer for this comment. While our study did not include DBS due to this study’s specific objectives and unavailable instrumentation, we recognize the importance of comparing microsampling techniques. Studies have highlighted key differences, particularly DBS's high variability in sample volume, which limits its utility for quantitative antibody measurements (1). Additionally, DBS workflows require manual or semi-automated processing steps, such as sample punching, centrifugation, and buffer extraction, increasing complexity and cost (2). In contrast, VAMS allows precise, standardized blood collection via capillary action (3), streamlining analysis by eliminating additional processing steps. Its compatibility with high-throughput platforms in the context of antibody detection has been shown (1, 4, 5), while minimizing hematocrit-related variations (3). We have added this background information to the manuscript introduction section, lines: 60-71. Additionally, we have included in the discussion a reflection for future research aimed at comparing VAMS with DBS, lines 348-350: ‘While the VAMS-based microsampling device presents a promising alternative to venipuncture, a direct comparison with other conventional microsampling methods, such as DBS, could yield valuable insights, and future research assessing its cost-effectiveness would be beneficial.’

We appreciate the reviewer’s insights and hope this clarification is helpful.

5. Manuscript Revision: For the manuscript to be considered for publication, the authors must address all the aforementioned remarks and rewrite their manuscript accordingly.

We thank the reviewer for the comments provided. We have tried to address the reviewer’s comments to the best of our ability and have adapted the manuscript accordingly.

**Reviewer #2:** COVID-19, caused by severe acute respiratory syndrome coronavirus 2 (SARS-CoV-2), has posed a number of challenges to the medical and scientific community in terms of medical management and, in particular, diagnosis. The aim is to diagnose patients so that they can be managed and to establish epidemiological surveillance. In order to reach a wider target group, it was important to validate new diagnostic methods and strategies.

This is the background to the study "Sensitive and feasible SARS-CoV-2 antibody detection from blood and saliva self-microsamples", the main objective of which was to evaluate the sensitivity and feasibility of the Mitra VAMS device for self-collection of finger prick blood and saliva to measure anti-SARS-CoV-2 antibodies using a high throughput electrochemiluminescence-based SARS-CoV-2 spike (S) antibody assay.

Strengths and weaknesses
The research was approved by the ethics committee and all the participants provided written informed consent. The article is well organized and largely follows the journal's manuscript guidelines. Immunoassays for the detection and monitoring of SARS-CoV-2 have been shown to be effective in saliva in previous studies. The originality of this study is that it allows us to evaluate the combination of self-sampling tools calibrated on different matrices, saliva and blood, with sensitive serological tests (Roche Elecsys anti-SARS-CoV-2 immunoassay) and high-throughput instruments (Roche Cobas 6000 e601).
We agree with the findings that this strategy would be effective in a pandemic context, where rapid diagnosis for early treatment is a key factor in epidemic control. This was an enormous challenge during the COVID-19 pandemic in a context of restricted circulation and individual safety.
However, “Agreement was better between serum and professionally collected capillary blood (κ=0.88, CI: 0.66-1, P<.001) with 1 (1.0%) discordant sample, compared to self-collected capillary blood (κ=0.74, CI: 0.39-1, P<.001) with 2 (2.0%) discordant samples”.
These results highlight the need for more training in the use of self-testing devices to ensure quality results. This needs to be emphasized in the text, although the data are available in the appendices.

We thank the reviewer for pointing this out. We have revised manuscript lines 322-326 to highlight the need for training in self-sampling techniques. The text now reads as follows: “We observed significant intra-rater agreement between capillary blood and serum samples in participants with unknown antibody status, ranging from good (κ=0.74) for those collected by study participants to almost perfect (κ=0.88) for professionally collected samples. These findings emphasize the importance of additional training in self-sampling techniques to ensure accuracy and reliability.” Additionally, we added to the abstract conclusions the following sentence: ‘To ensure accuracy and reliability, additional training in self-sampling techniques may be essential.’

Reviewer #3: I read the manuscript with great interest. The study focuses on the choice of sample and sampling methods, which are very critical in clinical laboratory diagnosis, in particular during a pandemic, quality laboratory diagnosis, and easy-to-collect samples are essential parts of pandemic containment. Thus, as part of preparedness for the coming pandemic, the study is important, despite the fact that they have employed an expensive technology that will make it difficult for resource-limited settings to benefit from the technology.

My concerns about the study:

1. The authors used high-throughput electrochemiluminescence technology, a very expensive one, for the antibody analysis. How is this feasible for scalability?

We thank the reviewer for this comment. The Roche Elecsys platform is a well-established platform with excellent performance, particularly suited for our exploratory work around alternative sample types. The Mitra microsampler has been demonstrated to be compatible with various high-throughput alternatives that may be less costly, such as in-house ELISA and nano-immunoassays. For example, Kalish et al. successfully used the Mitra microsampler with an in-house ELISA to determine SARS-CoV-2 positivity in nearly 10,000 individuals in the U.S (4, 6). Similarly, Michielin et al. utilized a nano-immunoassay capable of processing up to 1,024 samples in parallel with minimal sample and reagent consumption, showing promise for cost-effective serological testing (5). Additionally, the Mitra microsampler has been validated for use in influenza studies with an in-house bead-based multiplex assay, further supporting its scalability for large-scale applications using alternative high-throughput platforms (1).

2. How could the authors talk about the cost-effectiveness of their method without comparing the prices of different versions of sample collection methods?
We thank the reviewer for this comment. We would like to clarify that our study does not claim cost-effectiveness, as this was not within the scope of our research. Further studies are needed to properly evaluate cost-effectiveness. However, we do recognize the potential for cost-saving.

In large-scale serosurveillance programs, cost-effectiveness extends beyond the direct cost of sample collection to include factors such as sample preparation, shipping, storage, and analysis (3, 5, 7). The method we discuss offers significant advantages in these areas, principally over venipuncture, including eliminating the need for specialized personnel for sample collection, cold-chain storage and shipping, reducing reagent consumption, and allowing seamless integration with semi-automated bioanalytical laboratory workflows (3). In this regard, VAMS may also present an advantage over other microsampling methods, such as dried blood spots, which typically require additional specialized equipment—such as a puncher, extra processing workstations, and a compatible immunoanalyzer platform, as demonstrated by Beyerl et al. (2).

Furthermore, high-throughput capacity further enhances cost efficiency, particularly when paired with scalable serological assays. While the Roche Elecsys is relatively costly, VAMS has been explored with alternative platforms (see response to Q1). Moreover, as noted by Beyerl et al., (2) Roche Elecsys platforms are widely in use and benefit from a well-established supply chain coverage, making them accessible to centralized laboratories worldwide. Ultimately, cost-effectiveness should be evaluated in terms of the overall balance between cost and efficiency, rather than direct pricing alone.

We have added a sentence in the discussion (lines 353-355) to acknowledge the value of conducting a cost-effectiveness analysis in future research: ‘While the VAMS-based microsampling device presents a promising alternative to venipuncture, a direct comparison with other conventional microsampling methods, such as DBS, could yield valuable insights, and future research assessing its cost-effectiveness would be beneficial.’

3. When it comes to result interpretation, my major concern comes from the study included small study participants, and they have a mixed population with natural infection and vaccination, which the authors also acknowledged. In addition to what they did, I expect the authors to disaggregate the study participants into three those with natural infection with no vaccination, those Vaccinated but not infected, and both infected and vaccinated, and re-do the analysis and see the test agreements between the groups.

We thank the reviewer for this comment. The reviewer requests an analysis on 1) naturally infected without vaccination (n=4); 2) no natural infection with vaccination (n=92); and 3) naturally infected with vaccination (n= 48) (Table 1).

Table 1.

|  | **COVID-19 Vaccination status** | | | **Total** |
| --- | --- | --- | --- | --- |
| **Past COVID-19 infection** | **No** | **Yes** | **Missing** |  |
| **No** | 3 | 92 | 0 | 95 |
| **Yes** | 4 | 48 | 1 | 53 |
| **Missing** | 0 | 1 | 0 | 1 |
| **Total** | 7 | 141 | 1 | 149 |

As reported in the demographics table of the manuscript, 141/149 participants were vaccinated, which makes further analyses by subgroup difficult. The small numbers in these categories do not allow us to formally measure agreement and its statistical significance. However, we present the disaggregated results as requested by the reviewer.

**Agreement between paired capillary blood and serum samples.**

1. **Naturally infected without vaccination**. The first grouping (1) results in kappa=1, however, this cannot be properly interpreted due to the low number of observations.

|  | **Serum** | | |  |
| --- | --- | --- | --- | --- |
| **Capillary blood** |  | **Positive** | **Negative** | **Total** |
|  |  |  |  |  |
| **Health professional** | **Positive** | 3 | 0 | 3 |
|  | **Negative** | 0 | 1 | 1 |
|  | **Total** |  |  | 4 |
|  |  |  |  |  |
| **Participants** | **Positive** | 3 | 0 | 3 |
|  | **Negative** | 0 | 1 | 1 |
|  | **Total** |  |  | 4 |

2. **No natural infection with vaccination**. The agreement in the second grouping (2) is not possible to measure due to the absence of any negative samples in serum:

|  | **Serum** | | |  |
| --- | --- | --- | --- | --- |
| **Capillary blood** |  | **Positive** | **Negative** | **Total** |
|  |  |  |  |  |
| **Health professional** | **Positive** | 91 | 0 | 91 |
|  | **Negative** | 1 | 0 | 1 |
|  | **Total** |  |  | 92 |
|  |  |  |  |  |
| **Participants** | **Positive** | 91 | 0 | 91 |
|  | **Negative** | 1 | 0 | 1 |
|  | **Total** |  |  | 92 |

3. **Naturally infected with vaccination**. The agreement in the third grouping (3) is not possible to measure due to the absence of any negative samples in serum:

|  | **Serum** | | |  |
| --- | --- | --- | --- | --- |
| **Capillary blood** |  | **Positive** | **Negative** | **Total** |
|  |  |  |  |  |
| **Health professional** | **Positive** | 48 | 0 | 48 |
|  | **Negative** | 0 | 0 | 0 |
|  | **Total** |  |  | 48 |
|  |  |  |  |  |
| **Participants** | **Positive** | 48 | 0 | 48 |
|  | **Negative** | 0 | 0 | 0 |
|  | **Total** |  |  | 48 |

Although we cannot measure agreement, we show that there are no differences in the frequency of observations in antibody test results under any of the three groupings.

**Editor’s comments**

Dear Dr. Morales,

Thank you for submitting your manuscript to PLOS ONE. After careful consideration, we find that your work has merit and aligns with the scope of our journal. However, it does not fully meet PLOS ONE's publication criteria in its current form. We would like to invite you to submit a revised version of your manuscript, addressing the points raised during the review process.

While Reviewer 2 has recommended "acceptance", he has also provided comments that need to be addressed to ensure the manuscript meets the highest standards of clarity and rigor. We thank the Editor for this comment and this point has been addressed in the manuscript as requested by the reviewer.

Additionally, we kindly ask you to reconsider the title of your manuscript to ensure it accurately reflects its content and findings and to satisfy all reviewer's comment. We thank the Editor for pointing this out. The manuscript’s title has been revised considering the reviewer’s suggestion.

Please note that if your manuscript includes a video, it must be made publicly available upon publication. Kindly include a link to the video in the revised submission. We thank the Editor for highlighting this. A link to the English version of the video has now been included in the manuscript.

Journal Requirements:

When submitting your revision, we need you to address these additional requirements.

1. Please ensure that your manuscript meets PLOS ONE's style requirements. We have revised the style requirements of the Journal and the re-submitted manuscript and response to reviewers have been named as suggested.
2. We note that “S2_Appendix.pdf” in your submission contain copyrighted images. We require you to either (1) present written permission from the copyright holder to publish these figures specifically under the CC BY 4.0 license, or (2) remove the figures from your submission. A CC BY 4.0 license was requested. However, the manufacturer has inquired whether Plos One would be willing to grant a single use access since their instructional leaflets are updated.
3. We note that the grant information you provided in the ‘Funding Information’ and ‘Financial Disclosure’ sections do not match. This has been revised and the grant number corrected in the system.
4. In the online submission form, you indicated that “The data that support the findings of this study are not openly available due to reasons of sensitivity and are available from the corresponding author upon reasonable request. Data are located in controlled and secure access data storage at Heidelberg University Hospital. Video requests will be considered on a case-by-case basis due to reasons of sensitivity.” All relevant data are contained in the paper and supporting information files. Additionally, the disaggregated antibody test results have been included as S8 Table.
5. Please review your reference list to ensure that it is complete and correct. If you have cited papers that have been retracted, please include the rationale for doing so in the manuscript text, or remove these references and replace them with relevant current references. Any changes to the reference list should be mentioned in the rebuttal letter that accompanies your revised manuscript. References have been revised for accuracy and the following ones were removed and/or replaced:

- Freeman JD, Rosman LM, Ratcliff JD, Strickland PT, Graham DR, Silbergeld EK. State of the Science in Dried Blood Spots. Clin Chem. 2018;64(4):656-79. Reference does not address the information included in the introduction on the limited scalability of current antibody testing strategies due to the requirement for venipuncture and was therefore removed.
- Taffertshofer K, Walter M, Mackeben P, Kraemer J, Potapov S, Jochum S. Design and performance characteristics of the Elecsys anti-SARS-CoV-2 S assay. Front Immunol. 2022;13:1002576. Reference does not show the intended data about the clinical sensitivity and specificity of the Roche Elecsys assay as reported by the manufacturer and was therefore removed.
- Takei S, Ai T, Yamamoto T, Igawa G, Kanno T, Tobiume M, et al. Performance evaluation of the Roche Elecsys(R) Anti-SARS-CoV-2 immunoassays by comparison with neutralizing antibodies and clinical assessment. PLoS One. 2022;17(9):e0274181. Reference does not show the intended data about the clinical sensitivity and specificity of the Roche Elecsys assay as reported by the manufacturer and was therefore removed.
- Meyer B, Torriani G, Yerly S, Mazza L, Calame A, Arm-Vernez I, et al. Validation of a commercially available SARS-CoV-2 serological immunoassay. Clin Microbiol Infect. 2020;26(10):1386-94. Incorrect reference for a Euroimmune assay not used in this study.

This reference was exchanged for: Roche. Elecsys Anti-SARS-CoV-2 S. Package Insert 2020-09, V1.0; Material Numbers 09289267190 and 09289275190: Roche Diagnostics Deutschland GmbH; 2020 [Available from: <https://assets.cwp.roche.com/f/94122/x/37b1d9536f/factsheet-elecsys-anti-sars-cov-2-s_v3.pdf>.

This reference provides information for the assay’s clinical sensitivity and specificity as reported by the manufacturer.

**References**

1.	Wang J, Li D, Wiltse A, Emo J, Hilchey SP, Zand MS. Application of volumetric absorptive microsampling (VAMS) to measure multidimensional anti-influenza IgG antibodies by the mPlex-Flu assay. J Clin Transl Sci. 2019;3(6):332-43.

2.	Beyerl J, Rubio-Acero R, Castelletti N, Paunovic I, Kroidl I, Khan ZN, et al. A dried blood spot protocol for high throughput analysis of SARS-CoV-2 serology based on the Roche Elecsys anti-N assay. EBioMedicine. 2021;70:103502.

3.	Spooner N, Denniff P, Michielsen L, De Vries R, Ji QC, Arnold ME, et al. A device for dried blood microsampling in quantitative bioanalysis: overcoming the issues associated blood hematocrit. Bioanalysis. 2015;7(6):653-9.

4.	Klumpp-Thomas C, Kalish H, Drew M, Hunsberger S, Snead K, Fay MP, et al. Standardization of ELISA protocols for serosurveys of the SARS-CoV-2 pandemic using clinical and at-home blood sampling. Nat Commun. 2021;12(1):113.

5.	Michielin G, Arefi F, Puhach O, Bellon M, Sattonnet-Roche P, L'Huillier AG, et al. Clinical sensitivity and specificity of a high-throughput microfluidic nano-immunoassay combined with capillary blood microsampling for the identification of anti-SARS-CoV-2 Spike IgG serostatus. PLoS One. 2023;18(3):e0283149.

6.	Kalish H, Klumpp-Thomas C, Hunsberger S, Baus HA, Fay MP, Siripong N, et al. Undiagnosed SARS-CoV-2 seropositivity during the first 6 months of the COVID-19 pandemic in the United States. Sci Transl Med. 2021;13(601).

7.	Kok MGM, Fillet M. Volumetric absorptive microsampling: Current advances and applications. J Pharm Biomed Anal. 2018;147:288-96.
